# The Influence of (Dis)belief in Free Will on Immoral Behavior

**DOI:** 10.3389/fpsyg.2017.00020

**Published:** 2017-01-17

**Authors:** Emilie A. Caspar, Laurène Vuillaume, Pedro A. Magalhães De Saldanha da Gama, Axel Cleeremans

**Affiliations:** Consciousness, Cognition and Computation Group, Center for Research in Cognition and Neurosciences, ULB Neuroscience Institute, Université libre de BruxellesBruxelles, Belgium

**Keywords:** free will, moral behavior, sense of agency, coercion, social interactions, beliefs

## Abstract

One of the hallmarks of human existence is that we all hold beliefs that determine how we act. Amongst such beliefs, the idea that we are endowed with free will appears to be linked to prosocial behaviors, probably by enhancing the feeling of responsibility of individuals over their own actions. However, such effects appear to be more complex that one might have initially thought. Here, we aimed at exploring how induced disbeliefs in free will impact the sense of agency over the consequences of one’s own actions in a paradigm that engages morality. To do so, we asked participants to choose to inflict or to refrain from inflicting an electric choc to another participant in exchange of a small financial benefit. Our results show that participants who were primed with a text defending neural determinism – the idea that humans are a mere bunch of neurons guided by their biology – administered fewer shocks and were less vindictive toward the other participant. Importantly, this finding only held for female participants. These results show the complex interaction between gender, (dis)beliefs in free will and moral behavior.

## Introduction

People’s choice to act morally or not, that is, to carry out actions that are judged to be morally “right” or “wrong” is unsurprisingly shaped by many factors, ranging from genetics to education, from circumstances to religious beliefs. Amongst those many determinants, one is of particular interest because it subsumes the influence of many others: Our own beliefs about the extent to which we are free agents or, by contrast, our own beliefs that what we do is ultimately determined by factors upon which we have no control, such as the will of a god or the neural activity of our brains. The influence that belief or disbelief in free will exerts on behavior has attracted considerable interest recently (e.g., Schooler, 2010), in part because such beliefs are intimately connected with the judicial concept of responsibility. Indeed, our concept of responsibility, from a judicial point of view, is largely dependent on the general assumption that a defendant could have “done otherwise,” which itself presupposes that individuals are endowed with the freedom to choose their actions.

On this basis, it has thus been argued that reducing people’s belief in free will could weaken their beliefs in moral responsibility, thereby possibly modifying the moral character of their subsequent behavior. Congruently, a substantial body of scientific research has highlighted the prosocial benefits of believing in free will, as well as the negative effects of denying its existence (e.g., Wegner, 2002; Vohs and Schooler, 2008; Baumeister et al., 2009; Leotti et al., 2010; Stillman et al., 2010). For instance, Baumeister et al. (2009) suggested that people who believe in free will exhibit a higher prosocial and altruistic behavior, and Vohs and Schooler (2008) observed that participants who were primed with disbelief in free will cheated more often than a group of control participants. According to Baumeister (2008), believing in free will increases one’s motivation and willingness to make efforts, therefore resulting in higher self-control. This argument has been supported by recent electroencephalography studies showing that inducing disbelief in free will changes the neural processes underlying voluntary action (Rigoni et al., 2011) and post error adaptation (Rigoni et al., 2013, 2015). Behavioral studies have likewise shown that the feeling of agency, that is, the pre-reflective feeling of being in control of one’s own actions (Synofzik et al., 2008) was reduced when participants were primed with disbelief in free will (Aarts and van den Bos, 2011; Lynn et al., 2014).

Nonetheless, these “pro free will” arguments remain quite controversial (Miles, 2013). Some studies have indicated that believing in determinism may also have positive effects (Westlake and Paulhus, 2007; Krueger et al., 2014; Shariff et al., 2014). For instance, Shariff et al. (2014) showed that people who believe in determinism exhibit reduced retributive attitudes toward others. Congruently, Krueger et al. (2014) showed that people who strongly believe in free will tend to be more punitive. In the same line of thought, Westlake and Paulhus (2007) observed that people who scored high on free will tended to assign more severe sentences to offenders. However, these studies all used fictitious scenarios involving hypothetical protagonists. Typically, participants are asked to read vignettes that describe a criminal event and have to choose which punishment the protagonist of the story should receive. Thus, claims that (dis)belief in free will changes moral behavior have still to be corroborated in a more ecological paradigm.

To understand why people choose moral or immoral actions, one should use a more proper method which would consist in assessing how people experience their own responsibility while performing actions with different consequences toward others. The feeling of being responsible is strongly related to the sense of agency (SoA), that is, the feeling that we are the author of our own actions and their consequences in the external world (Moretto et al., 2011). Noteworthy, the SoA is not a unitary phenomenon, but is rather constituted of different conscious experiences of authorship. Recent conceptual developments have separated the feeling of agency from the judgment of agency (Synofzik et al., 2008). The judgment of agency refers to the explicit declaration that an outcome was (or not) caused by our own actions, while the feeling of agency refers to a pre-reflective sensorimotor experience of being the author of an action. Haggard et al. (2002) developed the intentional binding paradigm to assess this implicit feeling of agency. In this paradigm, participants have to estimate the delay between their action (i.e., a key press) and an outcome (i.e., a tone). If the movement is voluntary, the perceived time is shorter than in a condition in which the movement is involuntary (for instance, triggered by a TMS pulse over the motor cortex), suggesting that sense of being the author of an action modifies time perception, by reducing it (see also Wenke and Haggard, 2009).

In the present study, we aimed to explore how disbelief in free will impacts people’s behavior as well as their SoA over the consequences of their own actions. To investigate this issue, we used an experimental manipulation of belief in free will (Vohs and Schooler, 2008) together with the paradigm developed by Caspar et al. (2016). In the latter study, two participants (the “agent” and the “victim”) took turns to administer (or not) electrical shocks to each other in order to receive a small financial benefit. In two conditions, participants were free or coerced to deliver or not those electrical shocks.

We assessed to what extent (dis)belief in free will impacts or not three major effects: (1) the number of socially unacceptable actions that participants carry out, (2) participants’ vindictive behavior over their co-participant, and (3) participants’ SoA over those action effects. (1) According to previous studies, disbelief in free will increases antisocial behavior (e.g., Vohs and Schooler, 2008; Baumeister et al., 2009). Therefore, we expected that participants primed with disbelief in free will will inflict more painful electric shocks to the “victim” when they are free to decide which action to perform. (2) The literature has highlighted that disbelief in free will also has prosocial effects, mainly by reducing vindictive behaviors (e.g., Krueger et al., 2014; Shariff et al., 2014). In Caspar et al.’s (2016) study, participants who played the role of victim first tended to administer more shocks than they had received when subsequently playing the role of the agent, thus behaving vindictively. We expect to observe a reduction of this vindictive behavior when participants are primed with disbelief in free will. (3) Several studies have shown that intentional binding was reduced when people were primed with disbelief in free will, suggesting a reduced SoA (e.g., Aarts and van den Bos, 2011; Lynn et al., 2014). However, only neutral outcomes (i.e., neutral tones) were used in such studies; whereas it is reasonable to consider that this effect could be mediated by the valence associated with each particular outcome. Indeed, in daily life, the actions that we choose to carry out often have valenced effects, for instance by producing a benefit for someone else. In the same vein, negative action effects have been showed to reduce intentional binding (e.g., Yoshie and Haggard, 2013). Given that SoA is intimately linked to responsibility (Moretto et al., 2011), the effect of disbelief in free will could also modify the SoA over positive and negative action effects, which is of importance to understand what guides the decision to perform morally acceptable or unacceptable actions.

Importantly, the method of Vohs and Schooler (2008) proposes to compare two groups of participants, one primed with disbelief in free will and one primed with a neutral excerpt. However, the mere reading a neutral excerpt does not necessarily imply that participants in this group strongly belief in free will. In the present study, we therefore also assessed people’s core beliefs in free will and determinism prior to the manipulation, so as to investigate to what extent they mediate the observed effects.

## Materials and Methods

### Participants

Forty right-handed and naïve participants (20 females) were recruited by dyads. Dyads were not mixed across gender. During the recruitment procedure, we ensured that participants were not close friends or relatives by creating the triads ourselves based on their age or on the courses they attended. The sample size was based on estimated power size considering our experimental design. Participants received between €12 and €18 for their participation. The following exclusion criteria were decided in advance of the experiment: failure to produce temporal intervals covarying monotonically with actual action-tone interval, or failure to follow instructions. To identify participants for whom the action-tone intervals did not gradually increase with action-tone intervals, we performed a linear trend analysis with contrast coefficients -1, 0, 1 for the three delays we used. Three participants were excluded due to a non-significant linear trend analysis. Given that two of these participants belonged to the same group, we immediately re-tested an additional pair to avoid differences between groups. For the remaining participants, the mean age was 22.54 years old (*SD* = 2.83). Importantly, the mean age was the same regardless of gender (*p* > 0.9) or group (*p* > 0.3). All participants provided written informed consent prior to the experiment. The study was approved by the local ethical committee of the Université libre de Bruxelles (008/2016).

### Materials and Procedure

Several days (between 3 and 5) before their participation, participants completed two questionnaires online: The Interpersonal Reactivity Index (IRI, Davis, 1980) and the Free Will and Determinism scale (FAD-plus, Paulhus and Carey, 2011). The IRI is composed of four subscales. *Perspective taking* is the tendency to spontaneously adopt the psychological point of view of others. *Fantasy* taps respondents’ tendencies to transpose themselves imaginatively into the feelings and actions of fictitious characters in books, movies, and plays. *Empathic concern* assesses “other-oriented” feelings of sympathy and concern for unfortunate others. Finally, the *Personal distress* measures “self-oriented” feelings of personal anxiety and unease intense interpersonal settings (Davis, 1980). The FAD-plus is also composed of four subscales: the *Free will* subscale (e.g., “People have complete control over the decisions they make”), the *Scientific Determinism* subscale (e.g., “Your genes determine your future”), the *Fatalistic Determinism* subscale (e.g., “Fate already has a plan for everyone”) and the *Unpredictability* subscale (e.g., “People’s future cannot be predicted”). The order of the questionnaires was counterbalanced across participants.

Upon arrival at the laboratory, participants read an information sheet about the experimental procedure and the aim of the experiment. The two co-participants signed their individual consent forms simultaneously, ensuring that they were both aware of the other’s consent. In the consent form, participants were clearly asked to state whether or not they understood that they could withdraw from the experiment at any time without financial damage and without motivating their decision. Participants were equally divided in two groups: The Control group (five male dyads and five female dyads = 20 participants) and the No Free will group (five male dyads and five female dyads = 20 participants). In the Control group, participants were requested to read an excerpt from the book of Francis Crick (1994) *The Astonishing Hypothesis* that did not mention free will, but rather explained how psychologists tried to develop a method to assess consciousness (Vohs and Schooler, 2008). In the No Free will group, participants were given another excerpt from the same book that challenges the existence of free will by mentioning for instance that human behavior is totally determined by genetics. Both texts were translated in French by two native speakers. Participants were given a few minutes to carefully read the text and were informed they would have to write an abstract about the text at the end of the experimental session.

In each experimental condition, three persons were present in the room. Two participants engaged in two different roles (i.e., the “agent” and the “victim”), and the experimenter. The roles of the participants were assigned randomly, based on where participants chose to sit when they arrived in the room. One participant started by being the agent and the other participant the “victim.” Of thirty-nine participants, only one asked to start by playing the role of the “victim.” These roles were switched mid-way through the experiment, making the procedure fully reciprocal, similarly to the method used by Caspar et al. (2016). The agent and the “victim” were seated at a table, facing each other. A keyboard was placed between them, oriented toward the agent but visible by both. The experimental task ran on a computer located on the agent’s right side, with the screen visible only to the agent and to the experimenter. The agent was instructed to press a key on the keyboard at a time she chose after the start of the trial, using the right index finger. A tone occurred after the key press. The delay between key press and tone was set to vary randomly at 200, 500, and 800 ms. If a shock was delivered, the shock occurred at the same time as the tone, so as to avoid perceptual bias. The participants’ task was to estimate the delay between the agent’s key press and the tone. They were informed that the delay would vary randomly on a trial-by-trial basis, between 1 and 1,000 ms (they were reminded that 1,000 ms equals 1 s). Participants were also told (1) to make use of all possible numbers between 1 and 1,000, as appropriate, (2) to avoid restricting their answer space (i.e., not to keep using numbers falling between 100 and 200), and (3) to avoid rounding. Each participant received a paper sheet with 60 empty boxes in which to write their time estimates in each condition of the task. Participants’ answers were hidden from view of the other participants by a cardboard so as to avoid participants being biased by the other participants’ answers.

**FIGURE 1:**
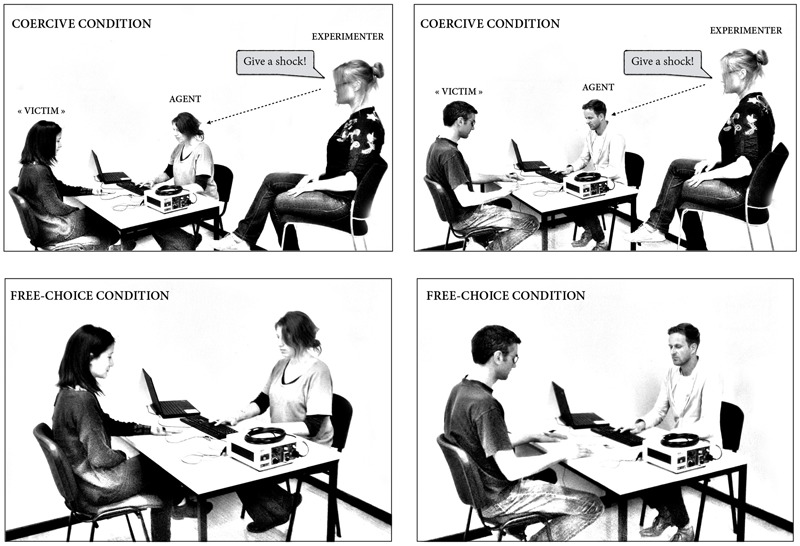
**Experimental set-up**.

Two experimental conditions followed a short training session, in which participants could practice the interval estimate procedure without any shocks or money to earn. All participants started with a specific amount of money, i.e., €12, for their participation. In the free-choice condition, agents were instructed that they could freely choose to increase their remuneration for the experiment by delivering a painful electric shock to the victim, using the appropriate key on the keyboard (“F” to deliver a shock and “H” to not deliver the shock). They were told that they were totally free to choose how to act. The agents earned €0.05 each time they decided to deliver a painful electric shock to the “victim.” They earned no money if they decided not to deliver a shock. In the coercive condition, the experimenter sat next to the agent and ordered her/him, on each trial, to deliver or not a painful electric shock to the victim (**Figure 1**). The experimenter ordered the agent to deliver a shock on 30/60 trials, in a random order.

There were 60 trials per condition (20 trials for each action-tone delay, in randomized order), resulting in a total of 240 trials (120 per role). Participants always performed the free-choice condition before the coercive condition to ensure that the number or shocks they choose to deliver in the free-choice condition would not be influenced by any other prior conditions.

Pain was delivered using a constant current stimulator (Digitimer DS7A) connected to two electrodes placed on the back of victims’ left hand, visible to the agent. Participants’ individual pain threshold was determined for the two participants after they had signed the consent form, before starting the experiment. This threshold was determined by increasing stimulation in steps of 1 mA, following the procedure described in Caspar et al. (2016). The mean stimulation level selected by this procedure was 31.85 mA (*SD* = 15.9, pulse duration: 200 s). Before starting the procedure, participants were told that the muscular stimulation would not cause any permanent damage or entail any risk of being burned.

At the end of the experiment, participants had to resume reading the text they had read at the beginning of the experimental session. In a post-session questionnaire, participants were invited to describe in a couple of words what they had felt during the experiment and what their thoughts were about this experiment. Finally, participants were paid separately based on their earned financial gain during the experiment.

## Results

To remember, we assessed whether or not disbelief in free will could influence: (1) the number of immoral actions performed, (2) vindictive behaviors, and (3) the implicit feeling of agency toward those events. We systematically explored the influence of the experimental group and gender on those dependant variables. In addition, we controlled whether or not the core beliefs of participants (as measured with the FAD-plus) could modify the results.

### Number of Shocks Freely Administered

**FIGURE 2:**
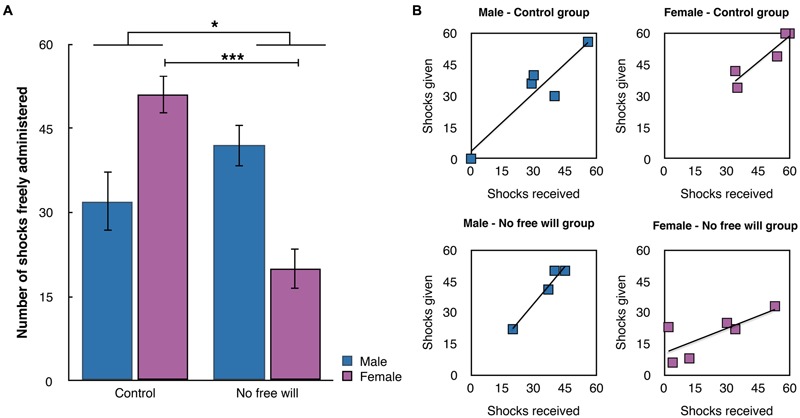
**Graphical representation of the number of shocks freely delivered (A)** and the vindictive behavior **(B)**. All tests were two-tailed. ^∗∗∗^ indicates a *p*-value ≤ 0.001 and ^∗^ indicates a *p*-value between 0.01 and 0.05.

When participants were agents, they freely chose to administer painful electric shocks to the victim in 36.29/60 trials (95% CI = 30.41–42.17, min 0, max 60). We conducted a factorial ANOVA with the number of shocks that agents freely delivered as the dependent variable and Gender (male, female) and Group (Control, No Free will) as fixed factors (see **Figure 2A**). The main effect of Gender was not significant (*p* > 0.7). The main effect of Group was significant [*F*(1,38) = 5.695, *p* < 0.025, η^2^ = 0.140]. Participants in the “No Free will” group freely delivered fewer shocks (30.90, 95% CI = 24.47–37.33) than participants in the Control group (41.45, 95% CI = 35.19–47.70). The interaction Gender × Group was significant [*F*(1,38) = 22.492, *p* < 0.001, η^2^ = 0.391]. Independent sample *t*-tests indicated that the number of shocks freely delivered did not differ according to group for male participants (*p* > 0.1), but differed significantly for female participants [*t*(18) = 6.459, *p* < 0.001, Cohen’s *d* = 6.55], suggesting that the procedure more strongly modified the behavior of female participants in comparison with male participants^1^ .

A previous study showed that the number of shocks administered was negatively correlated with the reported level of empathy of participants (Caspar et al., 2016). A factorial ANOVA showed that the empathy scores (IRI) did not differ between Groups (*p* > 0.5), but they did differ as a function of Gender [*F*(1,38) = 8.040, *p* = 0.008, η^2^ = 0.187]. Women (99.7, 95% CI = 94.5–104.9) reported being more empathetic than men (89.3, 95% CI = 84–94.6). The interaction Group × Gender was not significant (*p* > 0.4), suggesting that the reduced number of shocks delivered by female participants in the no free will group was not due to difference in empathy scores.

We additionally controlled whether or not the core beliefs of participants in each sub-group could constitute a confound in our results, given that basic beliefs differ between genders (Paulhus and Carey, 2011; Caspar et al., in press). We thus conducted a factorial ANOVA on each subscale of the FAD-plus. This showed that the interaction Group × Gender was never significant (all *p*s > 0.7). This result in turn suggests that the reduced number of shocks delivered by female participants in the no free will group was not due to differences in their core beliefs.

### Vindictive Behaviors

To assess vindictive behavior, we performed a linear regression with the number of shocks that participants who were victim first received as the independent variable and the number of shocks they gave when they were agents as the dependent variable (**Figure 2B**). In the Control group, we observed that the more shocks participants received when they were victims, the more shocks they administered when they were agents [*t*(9) = 8.149, *p* < 0.001, Beta = 0.945]. In the No Free will group, we observed a reduced but similar pattern of results [*t*(9) = 3.388, *p* = 0.01, Beta = 0.768]. When gender was taken into account, both male and female participants displayed vindictive behavior in the control group [*t*(4) = 4.338, *p* = 0.023, Beta = 0.929 – shocks received: 31, *SD* = 20.44 – shocks given: 32.40, *SD* = 20.51 and *t*(4) = 4.298, *p* = 0.023, Beta = 0.928, respectively – shocks received: 48.20, *SD* = 12.69 – shocks given: 49, *SD* = 11.35], while in the No Free will group, vindictive behavior was no longer significant for female participants (*p* > 0.08, Beta = 0.749) but remained significant for male participants [*t*(3) = 7.046, *p* = 0.020, Beta = 0.980 – shocks received: 35.5, *SD* = 10.84 – shocks given: 40.25, *SD* = 14.15]. The female participants in the No free will group gave less shocks than what they received when they were agents (shocks received: 22.50, *SD* = 19.95 – shocks given: 19.50, *SD* = 10.44). All other groups administered more shocks when they were agent than when they were victims.

We again examined whether the core beliefs of participants, as measured by the FAD-plus, could explain the differences that we observed between groups. We centered all predictor variables (Free will, Scientific Determinism, Fatalistic Determinism, and Unpredictability) before building the model. We observed that the core beliefs of participants did not influence those results. For female participants, the effect of group was always strongly significant (all *p*s < 0.01) and none of the predictor variables modified this result (all *p*s > 0.1). For male participants, we observed that their basic beliefs in Fatalistic Determinism influenced their vindictive behavior (*R*^2^ = 0.962, *p* = 0.023), irrespective of the group. Results showed that the higher male participants scored on Fatalistic Determinism, the less vindictive they were. For female participants, we observed that their basic beliefs in free will marginally influenced their vindictive behaviors (*R*^2^ = 0.896, *p* = 0.06) irrespective of the group. The higher female participants scored on Free will, the more vindictive they were.

### Interval Estimates

**FIGURE 3:**
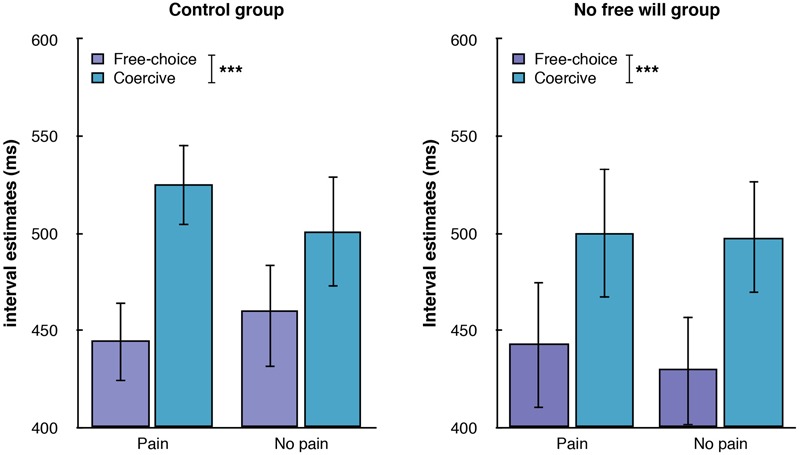
**Graphical representation of the interval estimates.** All tests were two-tailed. ^∗∗∗^ indicates a *p*-value ≤ 0.001.

We conducted a repeated-measures ANOVA with Condition (Free-Choice, Coercive) and Outcome (Harmful, Non harmful) as within-subject factors and Group (Control, No Free will) and Gender (Male, Female) as between-subject factors on agent’s interval estimates. Importantly, when participants freely administered the maximum (60/60 shocks, *N* = 5) or the minimum (0/60 shocks, *N* = 2) amount of shocks, their data were not taken into account in this full factorial ANOVA. The main effect of Condition was significant [*F*(1,28) = 28.870, *p* < 0.001, $\eta_{p}^{2}$ = 0.508], with coercion leading to longer interval estimates than free choice (494 ms, 95% CI: 453–536 and 443 ms, 95% CI: 401–485), see **Figure 3**. Importantly, none of the interactions of the factor Condition with the other factors were significant (all *p*s > 0.1). The main effect of Outcome was not significant (*p* > 0.2), nor was its interaction with the other factors (all *p*s > 0.2). Neither the main effect of Gender (*p* > 0.2) nor the main effect of Group (*p* > 0.9) were significant. Other triple interactions and the quadruple interaction also failed to reach significance (all *p*s > 0.078).

We performed a multiple linear regression to assess whether the subscales of the FAD-plus could predict better than the Group the “coercion effect,” that is, the difference between interval estimates in the free-choice condition and the coercive condition. We observed that the Unpredictability subscale was the best predictor of the coercion effect (*R*^2^ = 0.198, *p* = 0.017). The higher participants scored on Unpredictability, the higher the coercion effect was.

## Discussion

Here, we explored to what extent (dis)belief in free will could influence the moral decisions of participants in a task in which they were either free to choose or coerced to inflict an electric choc to their co-participants in exchange for a small financial benefit. Results mainly converge toward a prosocial benefit of disbelief in free will.

We first observed that participants in the No Free will group inflicted fewer shocks in the free-choice condition than participants in the Control group. However, this result appears to be mainly driven by gender. Indeed, while female participants inflicted fewer shocks in the No Free will group than in the Control group, this difference was not significant for male participants. In additional analyses, we examined whether or not this effect could be related to the reduction of vindictive behavior. We observed that the effect was similar if we only took into account participants who were agents first, thus eliminating the influence of role reversal. Moreover, we observed that the core beliefs of participants did not differ in these groups, and neither did their scores on empathy. Taken together, this suggests that the reduction of immoral behavior in the no free will group for female participants stems from the induced beliefs. The observed prosocial benefits of disbelief in free will may appear to go against the mainstream, since the literature mainly converged toward the prosocial benefits of believing in free will (e.g., Vohs and Schooler, 2008; Baumeister et al., 2009). However, numerous factors differed in our study, notably the social aspects associated with the presence of two co-participants who were aware that roles would be reversed at the middle of the experiment. Future work is required to explore this question more thoroughly.

Additionally, we observed that vindictive behavior was reduced for female participants in the no free will group compared to other sub-groups, and that the higher female participants scored on free will, the more vindictive they were. Importantly, our paradigm made it possible to investigate whether disbelief in free will influences the occurrence of vindictive behavior without the need to mention the notion of punishment to our participant, such as in previous studies. This tendency to behave vindictively is consistent with previous studies that showed that people who believe in determinism are less punitive and have reduced retributive attitudes toward others (e.g., Westlake and Paulhus, 2007; Krueger et al., 2014; Shariff et al., 2014). When people have to express a judgment about the morality of someone else’s behavior, their beliefs about the cause of these behaviors may greatly influence how they judge the severity of the act. Reducing people’s beliefs in free will might make them consider that individual responsibility is reduced, thus making them less retributive toward others.

Taken together, our results emphasize the importance of taking gender into account when studying beliefs. The gender of participants has indeed seldom been taken into account when assessing the influence of (dis)beliefs in free will on prosocial behaviors. Several studies that explored similar issues have often reported a discrepancy between males and females in their sample: female participants outnumber male participants, while importantly several studies pointed out they differ in their basic beliefs (Paulhus and Carey, 2011; Caspar et al., in press). Generally, men score higher in scientific determinism and women score higher in free will. It is thus plausible that inducing disbeliefs in free will has different effects on male and female participants. Therefore, some of the previous results could be mediated by such differences. The question of why female participants appear to be more influenced by the procedure remains pending. Several studies discussed gender difference in terms of factors, such as influenceability (e.g., Eagly, 1983), persuasibility (e.g., Janis and Field, 1959) or suggestibility (e.g., Page and Green, 2007), but such results seem unreliable (e.g., Pollard et al., 2004; Dienes et al., 2009). Therefore, future studies are needed to investigate the possible influence of further factors that could explain differences between male and female participants.

Contrary to previous studies (e.g., Lynn et al., 2014), we did not find that implicit SoA was higher in the control group compared to the no free will group. Rather we observed no statistical difference between the two groups in their interval estimates. In our paradigm, the implicit SoA was assessed in a moral context in which participants could decide to perform an action that was morally and socially acceptable or not, whereas in previous studies participants only had to judge the temporal delay between their action and a neutral tone without human interactions. In our daily life, however, our actions are seldom devoid of meaningful, valenced outcomes. When humans act, there is a purpose that is generally congruent with their goals or beneficial for themselves. Several studies have indeed shown that positive outcomes (Yoshie and Haggard, 2013) increase SoA. When participants have to focus on their own actions because the consequences have a deep impact on another person, it is possible that (dis)belief in free will becomes less significant for the measure associated with the intentional binding itself. Interestingly, we did not find that disbelief in free will affects SoA over morally inacceptable outcomes, such as initially considered. This is probably due to the combination of a negative outcome (i.e., delivering harm) and a positive outcome (i.e., earning additional money), which neutralized the effect (Takahata et al., 2012; Yoshie and Haggard, 2013). This might suggest that monetary gain can delude morality over negative outcomes, such as harming people, no matter people’s beliefs.

In the present study, we observed that the coercion effect was also present for male participants, thus extending previous results (Caspar et al., 2016). This suggests that the gender may have no direct effect on the mechanisms at work under coercion, but it does not eliminate the possibility that obedience is sensitive to such factors. Unexpectedly, we observed that part of the variance of the coercion effect was influenced by the unpredictability subscale. The higher participants scored on unpredictability (e.g., “Life is hard to predict because it is almost totally random”), the higher the coercion effect was. One possibility is that participants who consider that life is but a series of random events are more susceptible to let themselves go under coercion. However, this result has to be taken with caution. The equivalence between the size of the responses in terms of interval estimations and the feeling of agency has not been observed in a reliable manner.

## Conclusion

To conclude, we observed that disbelief in free will had a positive impact on the morality of decisions toward others. The present work extends previous research by showing that additional factors, such as gender, could influence the impact of (dis)belief in free will on prosocial and antisocial behaviors. Our results also showed that previous results relative to the (moral) context underlying the paradigm in use are not always replicated. The road toward progress in our understanding of how such beliefs influence human behavior remains long and arduous, but it clearly appears that both beliefs in free will and determinism can have positive impacts on moral-decision makings – a finding that challenges current thinking.

## Ethics Statement

The study was approved by the local ethical committee of the Université libre de Bruxelles (008/2016). All participants provided written informed consent prior to the experiment. On arrival at the experimental laboratory, participants read an information sheet about the experimental procedure and the aim of the experiment. The two co-participants signed their individual consent forms simultaneously, ensuring that they were both aware of the other’s consent.

## Author Contributions

EC developed the study concept. EC and LV created the study design and PG and AC provided critical comments. EC and LV ran the experiment. EC performed the data analysis and interpretation under the supervision of LV, PG, and AC. EC drafted the manuscript, and LV, PG, and AC provided critical revisions. All authors approved the final version of the manuscript for submission.

## Conflict of Interest Statement

The authors declare that the research was conducted in the absence of any commercial or financial relationships that could be construed as a potential conflict of interest.

## Footnotes

1 We replicated these analyses by taking into account only participants who were agents first. The interaction Group × Gender remained significant [*F*(1,18) = 11.581, *p* = 0.004, η^2^ = 0.436] and exhibited the same pattern of results.
